# Observation of ${{\mathrm {B}} ^+} \rightarrow {{\mathrm {J}/\uppsi }} 3{{\uppi } ^+} 2{{\uppi } ^-}$ and ${{\mathrm {B}} ^+} \rightarrow {\uppsi {(2\mathrm {S})}} {{\uppi } ^+} {{\uppi } ^+} {{\uppi } ^-}$ decays

**DOI:** 10.1140/epjc/s10052-017-4610-6

**Published:** 2017-02-06

**Authors:** R. Aaij, B. Adeva, M. Adinolfi, Z. Ajaltouni, S. Akar, J. Albrecht, F. Alessio, M. Alexander, S. Ali, G. Alkhazov, P. Alvarez Cartelle, A. A. Alves, S. Amato, S. Amerio, Y. Amhis, L. An, L. Anderlini, G. Andreassi, M. Andreotti, J. E. Andrews, R. B. Appleby, F. Archilli, P. d’Argent, J. Arnau Romeu, A. Artamonov, M. Artuso, E. Aslanides, G. Auriemma, M. Baalouch, I. Babuschkin, S. Bachmann, J. J. Back, A. Badalov, C. Baesso, S. Baker, W. Baldini, R. J. Barlow, C. Barschel, S. Barsuk, W. Barter, M. Baszczyk, V. Batozskaya, B. Batsukh, V. Battista, A. Bay, L. Beaucourt, J. Beddow, F. Bedeschi, I. Bediaga, L. J. Bel, V. Bellee, N. Belloli, K. Belous, I. Belyaev, E. Ben-Haim, G. Bencivenni, S. Benson, J. Benton, A. Berezhnoy, R. Bernet, A. Bertolin, C. Betancourt, F. Betti, M.-O. Bettler, M. van Beuzekom, Ia. Bezshyiko, S. Bifani, P. Billoir, T. Bird, A. Birnkraut, A. Bitadze, A. Bizzeti, T. Blake, F. Blanc, J. Blouw, S. Blusk, V. Bocci, T. Boettcher, A. Bondar, N. Bondar, W. Bonivento, I. Bordyuzhin, A. Borgheresi, S. Borghi, M. Borisyak, M. Borsato, F. Bossu, M. Boubdir, T. J. V. Bowcock, E. Bowen, C. Bozzi, S. Braun, M. Britsch, T. Britton, J. Brodzicka, E. Buchanan, C. Burr, A. Bursche, J. Buytaert, S. Cadeddu, R. Calabrese, M. Calvi, M. Calvo Gomez, A. Camboni, P. Campana, D. H. Campora Perez, L. Capriotti, A. Carbone, G. Carboni, R. Cardinale, A. Cardini, P. Carniti, L. Carson, K. Carvalho Akiba, G. Casse, L. Cassina, L. Castillo Garcia, M. Cattaneo, Ch. Cauet, G. Cavallero, R. Cenci, M. Charles, Ph. Charpentier, G. Chatzikonstantinidis, M. Chefdeville, S. Chen, S.-F. Cheung, V. Chobanova, M. Chrzaszcz, X. Cid Vidal, G. Ciezarek, P. E. L. Clarke, M. Clemencic, H. V. Cliff, J. Closier, V. Coco, J. Cogan, E. Cogneras, V. Cogoni, L. Cojocariu, G. Collazuol, P. Collins, A. Comerma-Montells, A. Contu, A. Cook, G. Coombs, S. Coquereau, G. Corti, M. Corvo, C. M. Costa Sobral, B. Couturier, G. A. Cowan, D. C. Craik, A. Crocombe, M. Cruz Torres, S. Cunliffe, R. Currie, C. D’Ambrosio, F. Da Cunha Marinho, E. Dall’Occo, J. Dalseno, P. N. Y. David, A. Davis, O. De Aguiar Francisco, K. De Bruyn, S. De Capua, M. De Cian, J. M. De Miranda, L. De Paula, M. De Serio, P. De Simone, C.-T. Dean, D. Decamp, M. Deckenhoff, L. Del Buono, M. Demmer, A. Dendek, D. Derkach, O. Deschamps, F. Dettori, B. Dey, A. Di Canto, H. Dijkstra, F. Dordei, M. Dorigo, A. Dosil Suárez, A. Dovbnya, K. Dreimanis, L. Dufour, G. Dujany, K. Dungs, P. Durante, R. Dzhelyadin, A. Dziurda, A. Dzyuba, N. Déléage, S. Easo, M. Ebert, U. Egede, V. Egorychev, S. Eidelman, S. Eisenhardt, U. Eitschberger, R. Ekelhof, L. Eklund, S. Ely, S. Esen, H. M. Evans, T. Evans, A. Falabella, N. Farley, S. Farry, R. Fay, D. Fazzini, D. Ferguson, A. Fernandez Prieto, F. Ferrari, F. Ferreira Rodrigues, M. Ferro-Luzzi, S. Filippov, R. A. Fini, M. Fiore, M. Fiorini, M. Firlej, C. Fitzpatrick, T. Fiutowski, F. Fleuret, K. Fohl, M. Fontana, F. Fontanelli, D. C. Forshaw, R. Forty, V. Franco Lima, M. Frank, C. Frei, J. Fu, E. Furfaro, C. Färber, A. Gallas Torreira, D. Galli, S. Gallorini, S. Gambetta, M. Gandelman, P. Gandini, Y. Gao, L. M. Garcia Martin, J. García Pardiñas, J. Garra Tico, L. Garrido, P. J. Garsed, D. Gascon, C. Gaspar, L. Gavardi, G. Gazzoni, D. Gerick, E. Gersabeck, M. Gersabeck, T. Gershon, Ph. Ghez, S. Gianì, V. Gibson, O. G. Girard, L. Giubega, K. Gizdov, V. V. Gligorov, D. Golubkov, A. Golutvin, A. Gomes, I. V. Gorelov, C. Gotti, E. Govorkova, M. Grabalosa Gándara, R. Graciani Diaz, L. A. Granado Cardoso, E. Graugés, E. Graverini, G. Graziani, A. Grecu, P. Griffith, L. Grillo, B. R. Gruberg Cazon, O. Grünberg, E. Gushchin, Yu. Guz, T. Gys, C. Göbel, T. Hadavizadeh, C. Hadjivasiliou, G. Haefeli, C. Haen, S. C. Haines, S. Hall, B. Hamilton, X. Han, S. Hansmann-Menzemer, N. Harnew, S. T. Harnew, J. Harrison, M. Hatch, J. He, T. Head, A. Heister, K. Hennessy, P. Henrard, L. Henry, J. A. Hernando Morata, E. van Herwijnen, M. Heß, A. Hicheur, D. Hill, C. Hombach, H. Hopchev, W. Hulsbergen, T. Humair, M. Hushchyn, N. Hussain, D. Hutchcroft, M. Idzik, P. Ilten, R. Jacobsson, A. Jaeger, J. Jalocha, E. Jans, A. Jawahery, F. Jiang, M. John, D. Johnson, C. R. Jones, C. Joram, B. Jost, N. Jurik, S. Kandybei, W. Kanso, M. Karacson, J. M. Kariuki, S. Karodia, M. Kecke, M. Kelsey, I. R. Kenyon, M. Kenzie, T. Ketel, E. Khairullin, B. Khanji, C. Khurewathanakul, T. Kirn, S. Klaver, K. Klimaszewski, S. Koliiev, M. Kolpin, I. Komarov, R. F. Koopman, P. Koppenburg, A. Kosmyntseva, A. Kozachuk, M. Kozeiha, L. Kravchuk, K. Kreplin, M. Kreps, P. Krokovny, F. Kruse, W. Krzemien, W. Kucewicz, M. Kucharczyk, V. Kudryavtsev, A. K. Kuonen, K. Kurek, T. Kvaratskheliya, D. Lacarrere, G. Lafferty, A. Lai, G. Lanfranchi, C. Langenbruch, T. Latham, C. Lazzeroni, R. Le Gac, J. van Leerdam, J.-P. Lees, A. Leflat, J. Lefrançois, R. Lefèvre, F. Lemaitre, E. Lemos Cid, O. Leroy, T. Lesiak, B. Leverington, Y. Li, T. Likhomanenko, R. Lindner, C. Linn, F. Lionetto, B. Liu, X. Liu, D. Loh, I. Longstaff, J. H. Lopes, D. Lucchesi, M. Lucio Martinez, H. Luo, A. Lupato, E. Luppi, O. Lupton, A. Lusiani, X. Lyu, F. Machefert, F. Maciuc, O. Maev, K. Maguire, S. Malde, A. Malinin, T. Maltsev, G. Manca, G. Mancinelli, P. Manning, J. Maratas, J. F. Marchand, U. Marconi, C. Marin Benito, P. Marino, J. Marks, G. Martellotti, M. Martin, M. Martinelli, D. Martinez Santos, F. Martinez Vidal, D. Martins Tostes, L. M. Massacrier, A. Massafferri, R. Matev, A. Mathad, Z. Mathe, C. Matteuzzi, A. Mauri, B. Maurin, A. Mazurov, M. McCann, J. McCarthy, A. McNab, R. McNulty, B. Meadows, F. Meier, M. Meissner, D. Melnychuk, M. Merk, A. Merli, E. Michielin, D. A. Milanes, M.-N. Minard, D. S. Mitzel, A. Mogini, J. Molina Rodriguez, I. A. Monroy, S. Monteil, M. Morandin, P. Morawski, A. Mordà, M. J. Morello, J. Moron, A. B. Morris, R. Mountain, F. Muheim, M. Mulder, M. Mussini, D. Müller, J. Müller, K. Müller, V. Müller, P. Naik, T. Nakada, R. Nandakumar, A. Nandi, I. Nasteva, M. Needham, N. Neri, S. Neubert, N. Neufeld, M. Neuner, A. D. Nguyen, T. D. Nguyen, C. Nguyen-Mau, S. Nieswand, R. Niet, N. Nikitin, T. Nikodem, A. Novoselov, D. P. O’Hanlon, A. Oblakowska-Mucha, V. Obraztsov, S. Ogilvy, R. Oldeman, C. J. G. Onderwater, J. M. Otalora Goicochea, A. Otto, P. Owen, A. Oyanguren, P. R. Pais, A. Palano, F. Palombo, M. Palutan, J. Panman, A. Papanestis, M. Pappagallo, L. L. Pappalardo, W. Parker, C. Parkes, G. Passaleva, A. Pastore, G. D. Patel, M. Patel, C. Patrignani, A. Pearce, A. Pellegrino, G. Penso, M. Pepe Altarelli, S. Perazzini, P. Perret, L. Pescatore, K. Petridis, A. Petrolini, A. Petrov, M. Petruzzo, E. Picatoste Olloqui, B. Pietrzyk, M. Pikies, D. Pinci, A. Pistone, A. Piucci, S. Playfer, M. Plo Casasus, T. Poikela, F. Polci, A. Poluektov, I. Polyakov, E. Polycarpo, G. J. Pomery, A. Popov, D. Popov, B. Popovici, S. Poslavskii, C. Potterat, E. Price, J. D. Price, J. Prisciandaro, A. Pritchard, C. Prouve, V. Pugatch, A. Puig Navarro, G. Punzi, W. Qian, R. Quagliani, B. Rachwal, J. H. Rademacker, M. Rama, M. Ramos Pernas, M. S. Rangel, I. Raniuk, F. Ratnikov, G. Raven, F. Redi, S. Reichert, A. C. dos Reis, C. Remon Alepuz, V. Renaudin, S. Ricciardi, S. Richards, M. Rihl, K. Rinnert, V. Rives Molina, P. Robbe, A. B. Rodrigues, E. Rodrigues, J. A. Rodriguez Lopez, P. Rodriguez Perez, A. Rogozhnikov, S. Roiser, A. Rollings, V. Romanovskiy, A. Romero Vidal, J. W. Ronayne, M. Rotondo, M. S. Rudolph, T. Ruf, P. Ruiz Valls, J. J. Saborido Silva, E. Sadykhov, N. Sagidova, B. Saitta, V. Salustino Guimaraes, C. Sanchez Mayordomo, B. Sanmartin Sedes, R. Santacesaria, C. Santamarina Rios, M. Santimaria, E. Santovetti, A. Sarti, C. Satriano, A. Satta, D. M. Saunders, D. Savrina, S. Schael, M. Schellenberg, M. Schiller, H. Schindler, M. Schlupp, M. Schmelling, T. Schmelzer, B. Schmidt, O. Schneider, A. Schopper, K. Schubert, M. Schubiger, M.-H. Schune, R. Schwemmer, B. Sciascia, A. Sciubba, A. Semennikov, A. Sergi, N. Serra, J. Serrano, L. Sestini, P. Seyfert, M. Shapkin, I. Shapoval, Y. Shcheglov, T. Shears, L. Shekhtman, V. Shevchenko, B. G. Siddi, R. Silva Coutinho, L. Silva de Oliveira, G. Simi, S. Simone, M. Sirendi, N. Skidmore, T. Skwarnicki, E. Smith, I. T. Smith, J. Smith, M. Smith, H. Snoek, M. D. Sokoloff, F. J. P. Soler, B. Souza De Paula, B. Spaan, P. Spradlin, S. Sridharan, F. Stagni, M. Stahl, S. Stahl, P. Stefko, S. Stefkova, O. Steinkamp, S. Stemmle, O. Stenyakin, S. Stevenson, S. Stoica, S. Stone, B. Storaci, S. Stracka, M. Straticiuc, U. Straumann, L. Sun, W. Sutcliffe, K. Swientek, V. Syropoulos, M. Szczekowski, T. Szumlak, S. T’Jampens, A. Tayduganov, T. Tekampe, G. Tellarini, F. Teubert, E. Thomas, J. van Tilburg, M. J. Tilley, V. Tisserand, M. Tobin, S. Tolk, L. Tomassetti, D. Tonelli, S. Topp-Joergensen, F. Toriello, E. Tournefier, S. Tourneur, K. Trabelsi, M. Traill, M. T. Tran, M. Tresch, A. Trisovic, A. Tsaregorodtsev, P. Tsopelas, A. Tully, N. Tuning, A. Ukleja, A. Ustyuzhanin, U. Uwer, C. Vacca, V. Vagnoni, A. Valassi, S. Valat, G. Valenti, A. Vallier, R. Vazquez Gomez, P. Vazquez Regueiro, S. Vecchi, M. van Veghel, J. J. Velthuis, M. Veltri, G. Veneziano, A. Venkateswaran, M. Vernet, M. Vesterinen, B. Viaud, D. Vieira, M. Vieites Diaz, H. Viemann, X. Vilasis-Cardona, M. Vitti, V. Volkov, A. Vollhardt, B. Voneki, A. Vorobyev, V. Vorobyev, C. Voß, J. A. de Vries, C. Vázquez Sierra, R. Waldi, C. Wallace, R. Wallace, J. Walsh, J. Wang, D. R. Ward, H. M. Wark, N. K. Watson, D. Websdale, A. Weiden, M. Whitehead, J. Wicht, G. Wilkinson, M. Wilkinson, M. Williams, M. P. Williams, M. Williams, T. Williams, F. F. Wilson, J. Wimberley, J. Wishahi, W. Wislicki, M. Witek, G. Wormser, S. A. Wotton, K. Wraight, K. Wyllie, Y. Xie, Z. Xing, Z. Xu, Z. Yang, H. Yin, J. Yu, X. Yuan, O. Yushchenko, K. A. Zarebski, M. Zavertyaev, L. Zhang, Y. Zhang, Y. Zhang, A. Zhelezov, Y. Zheng, A. Zhokhov, X. Zhu, V. Zhukov, S. Zucchelli

**Affiliations:** 1 Centro Brasileiro de Pesquisas Físicas (CBPF), Rio de Janeiro, Brazil; 2 Universidade Federal do Rio de Janeiro (UFRJ), Rio de Janeiro, Brazil; 3 Center for High Energy Physics, Tsinghua University, Beijing, China; 4 LAPP, Université Savoie Mont-Blanc, CNRS/IN2P3, Annecy-Le-Vieux, France; 5 Clermont Université, Université Blaise Pascal, CNRS/IN2P3, LPC, Clermont-Ferrand, France; 6 CPPM, Aix-Marseille Université, CNRS/IN2P3, Marseille, France; 7 LAL, Université Paris-Sud, CNRS/IN2P3, Orsay, France; 8 LPNHE, Université Pierre et Marie Curie, Université Paris Diderot, CNRS/IN2P3, Paris, France; 9 I. Physikalisches Institut, RWTH Aachen University, Aachen, Germany; 10 Fakultät Physik, Technische Universität Dortmund, Dortmund, Germany; 11 Max-Planck-Institut für Kernphysik (MPIK), Heidelberg, Germany; 12 Physikalisches Institut, Ruprecht-Karls-Universität Heidelberg, Heidelberg, Germany; 13 School of Physics, University College Dublin, Dublin, Ireland; 14 Sezione INFN di Bari, Bari, Italy; 15 Sezione INFN di Bologna, Bologna, Italy; 16 Sezione INFN di Cagliari, Cagliari, Italy; 17 Sezione INFN di Ferrara, Ferrara, Italy; 18 Sezione INFN di Firenze, Florence, Italy; 19 Laboratori Nazionali dell’INFN di Frascati, Frascati, Italy; 20 Sezione INFN di Genova, Genova, Italy; 21 Sezione INFN di Milano Bicocca, Milan, Italy; 22 Sezione INFN di Milano, Milan, Italy; 23 Sezione INFN di Padova, Padova, Italy; 24 Sezione INFN di Pisa, Pisa, Italy; 25 Sezione INFN di Roma Tor Vergata, Rome, Italy; 26 Sezione INFN di Roma La Sapienza, Rome, Italy; 27 Henryk Niewodniczanski Institute of Nuclear Physics Polish Academy of Sciences, Kraków, Poland; 28 Faculty of Physics and Applied Computer Science, AGH, University of Science and Technology, Kraków, Poland; 29 National Center for Nuclear Research (NCBJ), Warsaw, Poland; 30 Horia Hulubei National Institute of Physics and Nuclear Engineering, Bucharest-Magurele, Romania; 31 Petersburg Nuclear Physics Institute (PNPI), Gatchina, Russia; 32 Institute of Theoretical and Experimental Physics (ITEP), Moscow, Russia; 33 Institute of Nuclear Physics, Moscow State University (SINP MSU), Moscow, Russia; 34 Institute for Nuclear Research of the Russian Academy of Sciences (INR RAN), Moscow, Russia; 35 Yandex School of Data Analysis, Moscow, Russia; 36 Budker Institute of Nuclear Physics (BINP SB RAS), Novosibirsk, Russia; 37 Institute for High Energy Physics (IHEP), Protvino, Russia; 38 ICCUB, Universidad de Santiago de Compostela, Santiago de Compostela, Spain; 39 Universitat de Barcelona, Barcelona, Spain; 40 European Organization for Nuclear Research (CERN), Geneva, Switzerland; 41 Ecole Polytechnique Fédérale de Lausanne (EPFL), Lausanne, Switzerland; 42 Physik-Institut, Universität Zürich, Zurich, Switzerland; 43 Nikhef National Institute for Subatomic Physics, Amsterdam, The Netherlands; 44 Nikhef National Institute for Subatomic Physics, VU University Amsterdam, Amsterdam, The Netherlands; 45 NSC Kharkiv Institute of Physics and Technology (NSC KIPT), Kharkiv, Ukraine; 46 Institute for Nuclear Research of the National Academy of Sciences (KINR), Kyiv, Ukraine; 47 University of Birmingham, Birmingham, UK; 48 H.H. Wills Physics Laboratory, University of Bristol, Bristol, UK; 49 Cavendish Laboratory, University of Cambridge, Cambridge, UK; 50 Department of Physics, University of Warwick, Coventry, UK; 51 STFC Rutherford Appleton Laboratory, Didcot, UK; 52 School of Physics and Astronomy, University of Edinburgh, Edinburgh, UK; 53 School of Physics and Astronomy, University of Glasgow, Glasgow, UK; 54 Oliver Lodge Laboratory, University of Liverpool, Liverpool, UK; 55 Imperial College London, London, UK; 56 School of Physics and Astronomy, University of Manchester, Manchester, UK; 57 Department of Physics, University of Oxford, Oxford, UK; 58 Massachusetts Institute of Technology, Cambridge, MA USA; 59 University of Cincinnati, Cincinnati, OH USA; 60 University of Maryland, College Park, MD USA; 61 Syracuse University, Syracuse, NY USA; 62 Pontifícia Universidade Católica do Rio de Janeiro (PUC-Rio), Rio de Janeiro, Brazil; 63 University of Chinese Academy of Sciences, Beijing, China; 64 Institute of Particle Physics, Central China Normal University, Wuhan, Hubei China; 65 Departamento de Fisica, Universidad Nacional de Colombia, Bogotá, Colombia; 66 Institut für Physik, Universität Rostock, Rostock, Germany; 67 National Research Centre Kurchatov Institute, Moscow, Russia; 68 Instituto de Fisica Corpuscular (IFIC), Universitat de Valencia-CSIC, Valencia, Spain; 69 Van Swinderen Institute, University of Groningen, Groningen, The Netherlands

## Abstract

The decays ${{\mathrm {B}} ^+} \rightarrow {{\mathrm {J}/\uppsi }} 3{{\uppi } ^+} 2{{\uppi } ^-}$ and ${{\mathrm {B}} ^+} \rightarrow {\uppsi {(2\mathrm {S})}} {{\uppi } ^+} {{\uppi } ^+} {{\uppi } ^-}$ are observed for the first time using a data sample corresponding to an integrated luminosity of 3.0 fb$^{-1}$, collected by the LHCb experiment in proton–proton collisions at the centre-of-mass energies of 7 and 8$\mathrm {\,TeV}$. The branching fractions relative to that of ${{\mathrm {B}} ^+} \rightarrow {\uppsi {(2\mathrm {S})}} {{\mathrm {K}} ^+}$ are measured to be

$$\begin{aligned} \dfrac{{\mathcal {B}} ( {{\mathrm {B}} ^+} \rightarrow {{\mathrm {J}/\uppsi }} 3{{\uppi } ^+} 2{{\uppi } ^-} )}{{\mathcal {B}} ( {{\mathrm {B}} ^+} \rightarrow {\uppsi {(2\mathrm {S})}} {{\mathrm {K}} ^+} )}= & {} ( 1.88\!\pm \! 0.17\!\pm \! 0.09)\!\times \! 10^{-2}, \\ \dfrac{{\mathcal {B}} ( {{\mathrm {B}} ^+} \rightarrow {\uppsi {(2\mathrm {S})}} {{\uppi } ^+} {{\uppi } ^+} {{\uppi } ^-} )}{{\mathcal {B}} ( {{\mathrm {B}} ^+} \rightarrow {\uppsi {(2\mathrm {S})}} {{\mathrm {K}} ^+} )}= & {} ( 3.04\!\pm \!0.50\!\pm \!0.26)\!\times \! 10^{-2}, \end{aligned}$$

where the first uncertainties are statistical and the second are systematic.

## Introduction

The ${\mathrm {B}} ^+$  meson is a bound state of a heavy ${\overline{{\mathrm {b}}}}$ quark and a ${\mathrm {u}}$ quark, with well known properties and a large number of decay modes [1], but little is known about decays of ${{\mathrm {B}} ^+}$ mesons to a ${\mathrm {J}/\uppsi }$ meson plus a large number of light hadrons. The ${{\mathrm {B}} ^+} \rightarrow {{\mathrm {J}/\uppsi }} 3{{\uppi } ^+} 2{{\uppi } ^-}$ decay channel is of particular interest, since it is one of the highest multiplicity final states currently experimentally accessible. Evidence for the corresponding decay of the ${\mathrm {B}} _{\mathrm {c}} ^+$ meson has recently been reported by the LHCb collaboration [2], with the measured branching fraction and qualitative behaviour of the multipion system consistent with expectations from QCD factorisation [3, 4]. In this scheme, the ${{\mathrm {B}} _{\mathrm {c}} ^+} \rightarrow {{\mathrm {J}/\uppsi }} 3{{\uppi } ^+} 2{{\uppi } ^-}$ decay is characterized by the form factors of the ${{\mathrm {B}} _{\mathrm {c}} ^+} \rightarrow {{\mathrm {J}/\uppsi }} \mathrm {W} ^+$ transition and the spectral functions for the conversion of the $\mathrm {W} ^+$ boson into light hadrons [5–8]. Different decay topologies contribute to decays of ${\mathrm {B}} ^+$  mesons into charmonia and light hadrons, affecting the dynamics of the multipion system and enabling the role of factorisation in ${\mathrm {B}} ^+$  meson decays to be probed.

This paper describes an analysis of ${{\mathrm {B}} ^+} \rightarrow {{\mathrm {J}/\uppsi }} 3{{\uppi } ^+} 2{{\uppi } ^-}$ decays, including decays to the same final state that proceed through an intermediate $\uppsi {(2\mathrm {S})}$  resonance. Charge-conjugate modes are implied throughout the paper. The ratios of the branching fractions for each of these decays to that of the normalisation decay ${{\mathrm {B}} ^+} \rightarrow {\uppsi {(2\mathrm {S})}} {{\mathrm {K}} ^+}$,

1 $$\begin{aligned} R_{5\pi }&\equiv \frac{{\mathcal {B}} ({{\mathrm {B}} ^+} \rightarrow {{\mathrm {J}/\uppsi }} 3{{\uppi } ^+} 2{{\uppi } ^-} )}{{\mathcal {B}} ({{\mathrm {B}} ^+} \rightarrow {\uppsi {(2\mathrm {S})}} {{\mathrm {K}} ^+} )},\nonumber \\ R_{{\uppsi {(2\mathrm {S})}}}&\equiv \frac{{\mathcal {B}} ({{\mathrm {B}} ^+} \rightarrow {\uppsi {(2\mathrm {S})}} {{\uppi } ^+} {{\uppi } ^+} {{\uppi } ^-} )}{{\mathcal {B}} ({{\mathrm {B}} ^+} \rightarrow {\uppsi {(2\mathrm {S})}} {{\mathrm {K}} ^+} )}, \end{aligned}$$

are measured, where the ${\uppsi {(2\mathrm {S})}}$ meson is reconstructed in the ${{\mathrm {J}/\uppsi }} {{\uppi } ^+} {{\uppi } ^-}$ final state and the ${{\mathrm {J}/\uppsi }}$ meson is reconstructed in its dimuon decay channel. In addition, a search for intermediate resonances in the multipion system is performed and a phase-space model is compared to the data and to the predictions from QCD factorisation [3–8]. The results are based on ${\mathrm {p}} {\mathrm {p}}$ collision data corresponding to an integrated luminosity of 1.0 and 2.0 fb$^{-1}$ collected by the LHCb experiment at centre-of-mass energies of $\sqrt{s}=7$ and $8\mathrm {\,TeV}$, respectively.

## Detector and simulation

The LHCb detector [9, 10] is a single-arm forward spectrometer covering the pseudorapidity range $2<\eta <5$, designed for the study of particles containing $\mathrm {b}$ or $\mathrm {c}$  quarks. The detector includes a high-precision tracking system consisting of a silicon-strip vertex detector surrounding the ${\mathrm {p}} {\mathrm {p}}$ interaction region, a large-area silicon-strip detector located upstream of a dipole magnet with a bending power of about $4{\mathrm {\,Tm}}$, and three stations of silicon-strip detectors and straw drift tubes placed downstream of the magnet. The tracking system provides a measurement of momentum, $p$, of charged particles with a relative uncertainty that varies from 0.5% at low momentum to 1.0% at 200${\mathrm {\,GeV\!/}c}$. The minimum distance of a track to a primary vertex (PV), the impact parameter, is measured with a resolution of $(15+29/p_{\mathrm {T}}){\,\upmu \mathrm {m}}$, where $p_{\mathrm {T}}$ is the component of the momentum transverse to the beam in ${\mathrm {\,GeV\!/}c}$. Different types of charged hadrons are distinguished using information from two ring-imaging Cherenkov detectors (RICH). Photons, electrons and hadrons are identified by a calorimeter system consisting of scintillating-pad and preshower detectors, an electromagnetic calorimeter and a hadronic calorimeter. Muons are identified by a system composed of alternating layers of iron and multiwire proportional chambers.

The online event selection is performed by a trigger [11], which consists of a hardware stage, based on information from the calorimeter and muon systems, followed by a software stage, which applies a full event reconstruction. The hardware trigger selects muon candidates with $p_{\mathrm {T}} >1.48\,(1.76){\mathrm {\,GeV\!/}c}$ or pairs of opposite-sign muon candidates with a requirement that the product of the muon transverse momenta is larger than $1.7\,(2.6)\,\mathrm {GeV}^2/c^2$ for data collected at $\sqrt{s}=7\,(8)\mathrm {\,TeV}$. The subsequent software trigger is composed of two stages, the first of which performs a partial event reconstruction, while full event reconstruction is done at the second stage. In the software trigger the invariant mass of well-reconstructed pairs of oppositely charged muons forming a good-quality two-track vertex is required to exceed 2.7${\mathrm {\,GeV\!/}c^2}$, and the two-track vertex is required to be significantly displaced from all PVs.

The analysis technique reported below is validated using simulated events. In the simulation, ${\mathrm {p}} {\mathrm {p}}$ collisions are generated using Pythia [12, 13] with a specific LHCb configuration [14]. Decays of hadronic particles are described by EvtGen  [15], in which final-state radiation is generated using Photos  [16]. A model assuming QCD factorisation is implemented to generate the decays ${{\mathrm {B}} ^+} \rightarrow {{\mathrm {J}/\uppsi }} 3{{\uppi } ^+} 2{{\uppi } ^-}$ and ${{\mathrm {B}} ^+} \rightarrow {\uppsi {(2\mathrm {S})}} {{\uppi } ^+} {{\uppi } ^+} {{\uppi } ^-}$ [5]. The interaction of the generated particles with the detector and its response are implemented using the Geant4 toolkit [17, 18] as described in Ref. [19].

## Candidate selection

The decays ${{\mathrm {B}} ^+} \rightarrow {{\mathrm {J}/\uppsi }} 3{{\uppi } ^+} 2{{\uppi } ^-}$, ${{\mathrm {B}} ^+} \rightarrow {\uppsi {(2\mathrm {S})}} {{\uppi } ^+} {{\uppi } ^+} {{\uppi } ^-}$ and ${{\mathrm {B}} ^+} \rightarrow {\uppsi {(2\mathrm {S})}} {{\mathrm {K}} ^+}$ are reconstructed using the decay modes ${{\mathrm {J}/\uppsi }} \rightarrow {\upmu ^+\upmu ^-}$ and ${\uppsi {(2\mathrm {S})}} \rightarrow {{\mathrm {J}/\uppsi }} {{\uppi } ^+} {{\uppi } ^-}$ followed by ${{\mathrm {J}/\uppsi }} \rightarrow {\upmu ^+\upmu ^-}$. Similar selection criteria are applied to all channels in order to minimize the systematic uncertainties.

Muon, pion and kaon candidates are selected from well-reconstructed tracks and are identified using information from the RICH, calorimeter and muon detectors. Muon candidates are required to have a transverse momentum larger than $550{\mathrm {\,MeV\!/}c}$. Both pion and kaon candidates are required to have a transverse momentum larger than $250{\mathrm {\,MeV\!/}c}$ and momentum between 3.2 and $150{\mathrm {\,GeV\!/}c}$ to allow good particle identification. To reduce combinatorial background due to tracks from the ${\mathrm {p}} {\mathrm {p}}$ interaction vertex, only tracks that are inconsistent with originating from a PV are used.

Pairs of oppositely charged muons originating from a common vertex are combined to form ${{\mathrm {J}/\uppsi }} \rightarrow {\upmu ^+\upmu ^-}$ candidates. The mass of the dimuon combination is required to be between 3.020 and $3.135{\mathrm {\,GeV\!/}c^2}$. The asymmetric mass range around the known ${{\mathrm {J}/\uppsi }}$ meson mass [1] is chosen to include the low-mass tail due to final-state radiation.

To form a ${{\mathrm {B}} ^+}$  candidate, the selected ${\mathrm {J}/\uppsi }$ candidates are combined with $3{{\uppi } ^+} 2{{\uppi } ^-}$ or ${{\mathrm {K}} ^+} {{\uppi } ^+} {{\uppi } ^-}$ candidates for the signal and control decays, respectively. Each ${{\mathrm {B}} ^+}$  candidate is associated with the PV with respect to which it has the smallest $\chi ^2_{\text {IP}}$, which is defined as the difference in the vertex fit $\chi ^2$ of the PV with and without the particle under consideration. To improve the mass resolution, a kinematic fit [20] is applied. In this fit the mass of the ${\upmu ^+} {\upmu ^-}$ combination is fixed to the known ${\mathrm {J}/\uppsi }$ mass, and the ${{\mathrm {B}} ^+}$  candidate’s momentum vector is required to originate at the associated PV. A good-quality fit is required to further suppress combinatorial background. In addition, the measured decay time of the ${{\mathrm {B}} ^+}$ candidate, calculated with respect to the associated PV, is required to be larger than $200{\,\upmu \mathrm {m}}/c$, to suppress background from particles coming from the PV.

## Signal and normalisation yields

**Fig. 1:**
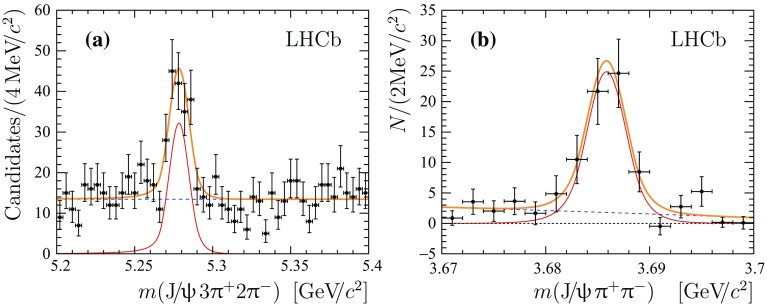
**a** Mass distribution of the selected ${{\mathrm {B}} ^+} \rightarrow {{\mathrm {J}/\uppsi }} 3{{\uppi } ^+} 2{{\uppi } ^-}$ candidates. **b** Sum of mass distributions for all background-subtracted ${{\mathrm {J}/\uppsi }} {{\uppi } ^+} {{\uppi } ^-}$ combinations. The total fit function is shown with thick *solid* (*orange*) *lines* and the signal contribution with thin *solid* (*red*) *lines*. The *dashed* (*blue*) *lines* represent the combinatorial background and non-resonance component for plots **a** and **b**, respectively

The mass distribution for selected ${{\mathrm {B}} ^+} \rightarrow {{\mathrm {J}/\uppsi }} 3{{\uppi } ^+} 2{{\uppi } ^-}$ candidates is shown in Fig. 1a. The signal yield is determined with an extended unbinned maximum likelihood fit to the distribution. The signal is modelled with a Gaussian function with power law tails on both sides [21], where the tail parameters are fixed from simulation and the peak position and the width of the Gaussian function are allowed to vary. The combinatorial background is modelled with a uniform distribution. No peaking backgrounds from misreconstructed or partially reconstructed decays of beauty hadrons are expected in the fit range. The resolution parameter obtained from the fit is found to be $6\pm 1{\mathrm {\,MeV\!/}c^2}$ and is in good agreement with the expectation from simulation. The observed signal yield is $139\pm 18$.

The statistical significance for the observed signal is determined as $\mathcal {S}_{\sigma }=\sqrt{-2\log \mathcal {L}_\mathrm {B}/\mathcal {L}_{\mathrm {S+B}}}$, where ${\mathcal {L}_{\mathrm {S+B}}}$ and ${\mathcal {L}_{\mathrm {B}}}$ denote the likelihood associated with the signal-plus-background and background-only hypothesis, respectively. The statistical significance of the ${{\mathrm {B}} ^+} \rightarrow {{\mathrm {J}/\uppsi }} 3{{\uppi } ^+} 2{{\uppi } ^-}$ signal is in excess of 10 standard deviations.

**Fig. 2:**
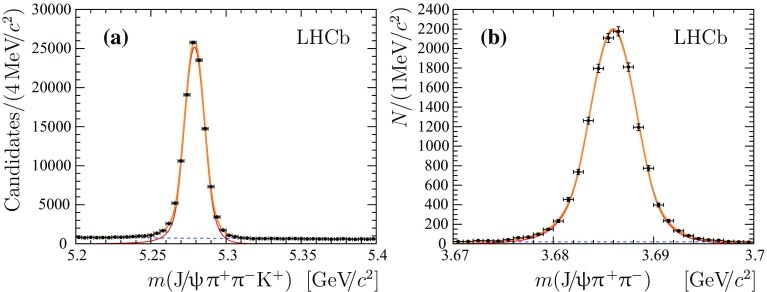
Mass distributions **a** of the selected ${{\mathrm {B}} ^+} \rightarrow {\uppsi {(2\mathrm {S})}} {{\mathrm {K}} ^+}$  candidates and **b** background-subtracted ${{\mathrm {J}/\uppsi }} {{\uppi } ^+} {{\uppi } ^-}$ combination. The total fit function is shown with thick *solid* (*orange*) *lines* and the signal contribution with thin *solid* (*red*) *lines*. The *dashed* (*blue*) *lines* represent the combinatorial background and non-resonance component for plots **a** and **b**, respectively

For the selected ${{\mathrm {B}} ^+}$  candidates, the existence of a resonant structure is searched for in the ${{\mathrm {J}/\uppsi }} {{\uppi } ^+} {{\uppi } ^-}$ combinations of final-state particles. There are six possible ${{\mathrm {J}/\uppsi }} {{\uppi } ^+} {{\uppi } ^-}$  combinations that can be formed from the ${{\mathrm {J}/\uppsi }} 3{{\uppi } ^+} 2{{\uppi } ^-}$ final state. The background-subtracted distribution of all six possible combinations in the narrow range around the known $\uppsi {(2\mathrm {S})}$  meson mass is shown in Fig. 1b, where each event enters six times. The *sPlot* technique is used for background subtraction [22] with the ${{\mathrm {J}/\uppsi }} 3{{\uppi } ^+} 2{{\uppi } ^-}$ mass as the discriminating variable. The signal yield of ${{\mathrm {B}} ^+} \rightarrow {\uppsi {(2\mathrm {S})}} [\rightarrow {{\mathrm {J}/\uppsi }} {{\uppi } ^+} {{\uppi } ^-} ]{{\uppi } ^+} {{\uppi } ^+} {{\uppi } ^-}$ is determined using an extended unbinned maximum likelihood fit to the background-subtracted ${{\mathrm {J}/\uppsi }} {{\uppi } ^+} {{\uppi } ^-}$ mass distribution. The $\uppsi {(2\mathrm {S})}$ component is modelled with a Gaussian function with power law tails on both sides, where the tail parameters are fixed from simulation. The non-resonant component is modelled with the phase-space shape multiplied by a linear function. The mass resolution obtained from the fit is $1.9\pm 0.3{\mathrm {\,MeV\!/}c^2}$, in good agreement with the expectation from simulation. The observed signal yield is $61\pm 10$.

The ${{\mathrm {B}} ^+} \rightarrow {\uppsi {(2\mathrm {S})}} [\rightarrow {{\mathrm {J}/\uppsi }} {{\uppi } ^+} {{\uppi } ^-} ]{{\mathrm {K}} ^+}$ decay is used as a normalisation channel for the measurements of the relative branching fractions. The mass distribution for selected ${{\mathrm {B}} ^+} \rightarrow {{\mathrm {J}/\uppsi }} {{\uppi } ^+} {{\uppi } ^-} {{\mathrm {K}} ^+}$ candidates is shown in Fig. 2a. An extended unbinned maximum likelihood fit to the distribution is performed using the model described above for the signal and an exponential function for the background. The mass resolution parameter obtained from the fit is $6.60\pm 0.02{\mathrm {\,MeV\!/}c^2}$, again in good agreement with the expectations from simulation. The background-subtracted mass distribution of the ${{\mathrm {J}/\uppsi }} {{\uppi } ^+} {{\uppi } ^-}$ system in the region of the $\uppsi {(2\mathrm {S})}$ mass is shown in Fig. 2b.

The signal yield of ${{\mathrm {B}} ^+} \rightarrow {\uppsi {(2\mathrm {S})}} [\rightarrow {{\mathrm {J}/\uppsi }} {{\uppi } ^+} {{\uppi } ^-} ]{{\mathrm {K}} ^+}$ is determined using an extended unbinned maximum likelihood fit to the ${{\mathrm {J}/\uppsi }} {{\uppi } ^+} {{\uppi } ^-}$ distribution, where the background is subtracted using the *sPlot* technique with the ${{\mathrm {J}/\uppsi }} {{\uppi } ^+} {{\uppi } ^-} {{\mathrm {K}} ^+}$ mass as the discriminating variable. The $\uppsi {(2\mathrm {S})}$ and the non-resonant components are modelled with the same functions used for the signal channel. The mass resolution obtained from the fit is $2.35\pm 0.02{\mathrm {\,MeV\!/}c^2}$. The signal yields are summarized in Table 1.

**Table 1:** Signal yields, *N*, of ${{\mathrm {B}} ^+}$ decay channels. Uncertainties are statistical only

Channel	*N* (${{\mathrm {B}} ^+}$)
${{\mathrm {B}} ^+} \rightarrow {{\mathrm {J}/\uppsi }} 3{{\uppi } ^+} 2{{\uppi } ^-}$	$139\pm 18$
${{\mathrm {B}} ^+} \rightarrow {\uppsi {(2\mathrm {S})}} [\rightarrow {{\mathrm {J}/\uppsi }} {{\uppi } ^+} {{\uppi } ^-} ]{{\uppi } ^+} {{\uppi } ^+} {{\uppi } ^-}$	$61\pm 10$
${{\mathrm {B}} ^+} \rightarrow {\uppsi {(2\mathrm {S})}} [\rightarrow {{\mathrm {J}/\uppsi }} {{\uppi } ^+} {{\uppi } ^-} ]{{\mathrm {K}} ^+}$	$13{,}554\pm 118$

**Fig. 3:**
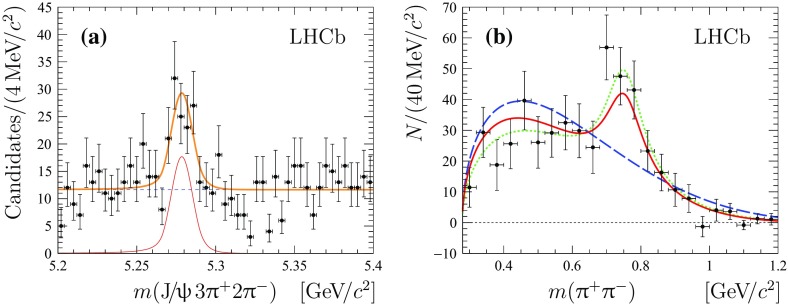
**a** Mass distribution of the selected ${{\mathrm {B}} ^+} \rightarrow {{\mathrm {J}/\uppsi }} 3{{\uppi } ^+} 2{{\uppi } ^-}$ candidates with the additional requirement of every ${{\mathrm {J}/\uppsi }} {{\uppi } ^+} {{\uppi } ^-}$ combination to be outside of $\pm 6{\mathrm {\,MeV\!/}c^2}$ around the known $\uppsi {(2\mathrm {S})}$  mass. The total fit function, the ${\mathrm {B}} ^+$  signal contribution and the combinatorial background are shown with thick *solid* (*orange*), *thin solid* (*red*) and *dashed* (*blue*) *lines*, respectively. **b** Sum of mass distributions for all possible background-subtracted ${{\uppi } ^+} {{\uppi } ^-}$ combinations. The factorisation-based model prediction is shown by a *solid* (*red*) *line*, and the expectation from the phase-space model is shown by a *dashed* (*blue*) *line*. The total fit function, shown with a *dotted* (*green*) *line*, is an incoherent sum of a relativistic Breit–Wigner function with the mean and natural width fixed to the known $\uprho ^{0}$ values and a phase-space function multiplied by a second-order polynomial

## Study of the multipion system

A search for intermediate light resonances is performed on the set of events which do not decay through the $\uppsi {(2\mathrm {S})}$  resonance. For this, the additional criterion that the mass of every ${{\mathrm {J}/\uppsi }} {{\uppi } ^+} {{\uppi } ^-}$  combination is outside $\pm 6{\mathrm {\,MeV\!/}c^2}$ around the known $\uppsi {(2\mathrm {S})}$  meson mass [1] is applied. The invariant-mass distribution for ${{\mathrm {B}} ^+} \rightarrow {{\mathrm {J}/\uppsi }} 3{{\uppi } ^+} 2{{\uppi } ^-}$ candidates selected with the veto on the $\uppsi {(2\mathrm {S})}$ resonance is shown in Fig. 3a. A clear peak, corresponding to the non-resonant decay ${{\mathrm {B}} ^+} \rightarrow {{\mathrm {J}/\uppsi }} 3{{\uppi } ^+} 2{{\uppi } ^-}$ decay is visible. The signal yield for this channel is determined using an extended unbinned maximum likelihood fit using the function described above. The observed signal yield is $80\pm 15$ with a statistical significance of 6.8 standard deviations.

**Fig. 4:**
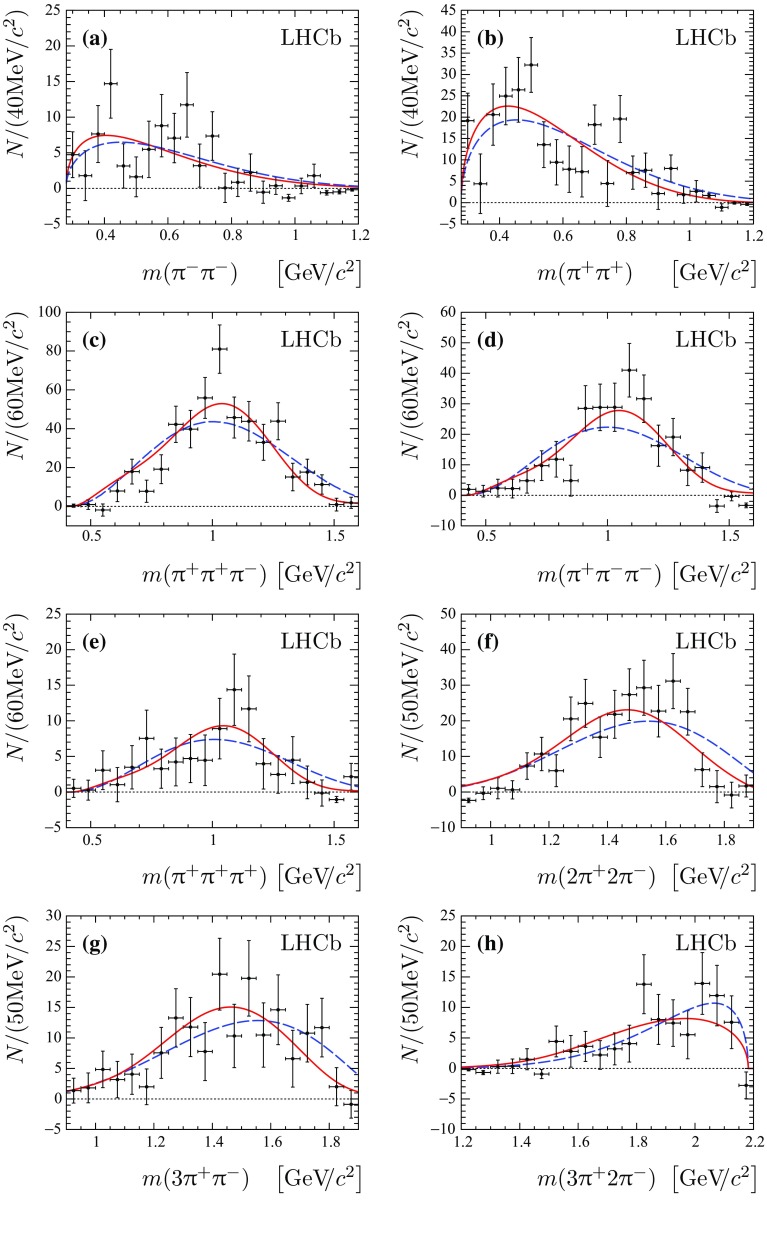
Distributions of **a** ${{\uppi } ^-} {{\uppi } ^-}$, **b** ${{\uppi } ^+} {{\uppi } ^+}$, **c** ${{\uppi } ^+} {{\uppi } ^+} {{\uppi } ^-}$, **d** ${{\uppi } ^+} {{\uppi } ^-} {{\uppi } ^-}$, **e** ${{\uppi } ^+} {{\uppi } ^+} {{\uppi } ^+}$, **f** $2{{\uppi } ^+} 2{{\uppi } ^-}$, **g** $3{{\uppi } ^+} {{\uppi } ^-}$ and **h** $3{{\uppi } ^+} 2{{\uppi } ^-}$ masses in the ${{\mathrm {B}} ^+} \rightarrow {{\mathrm {J}/\uppsi }} 3{{\uppi } ^+} 2{{\uppi } ^-}$ decay. The prediction from the factorisation-based model is shown by *solid* (*red*) *lines*, and the expectation from the phase-space model is shown by *dashed* (*blue*) *lines*

The resonance structure is investigated in the ${{\uppi } ^+} {{\uppi } ^-}$, ${{\uppi } ^+} {{\uppi } ^+}$, ${{\uppi } ^-} {{\uppi } ^-}$, ${{\uppi } ^+} {{\uppi } ^+} {{\uppi } ^-}$, ${{\uppi } ^+} {{\uppi } ^-} {{\uppi } ^-}$, ${{\uppi } ^+} {{\uppi } ^+} {{\uppi } ^+}$, $2{{\uppi } ^+} 2{{\uppi } ^-}$, $3{{\uppi } ^+} {{\uppi } ^-}$ and $3{{\uppi } ^+} 2{{\uppi } ^-}$ combinations of final-state particles using the *sPlot* technique, with the reconstructed ${{\mathrm {J}/\uppsi }} 3{{\uppi } ^+} 2{{\uppi } ^-}$ mass as the discriminating variable. The resulting background-subtracted mass distribution of all possible ${{\uppi } ^+} {{\uppi } ^-}$ combinations is shown in Fig. 3b, along with the theoretical predictions from the factorisation approach and the phase-space model [5–8]. A structure is seen that can be associated to the $\uprho ^{0}$ meson. The distribution is fitted with a sum of a relativistic Breit–Wigner function with the mean and natural width fixed to the known $\uprho ^{0}$ values plus a phase-space shape multiplied by a second-order polynomial. No significant narrow structures are observed for other multipion combinations. The distributions for all other combinations of pions are compared with predictions of both a factorisation approach and a phase-space model, as shown in Fig. 4. For all fits the $\chi ^2$ per degree of freedom, $\chi ^2/\mathrm {ndf}$, is given in Table 2. The prediction from the factorisation approach is found to be in somewhat better agreement with the data than that from the phase-space model, giving better $\chi ^2/\mathrm {ndf}$ values for eight out of nine distributions examined.

**Fig. 5:**
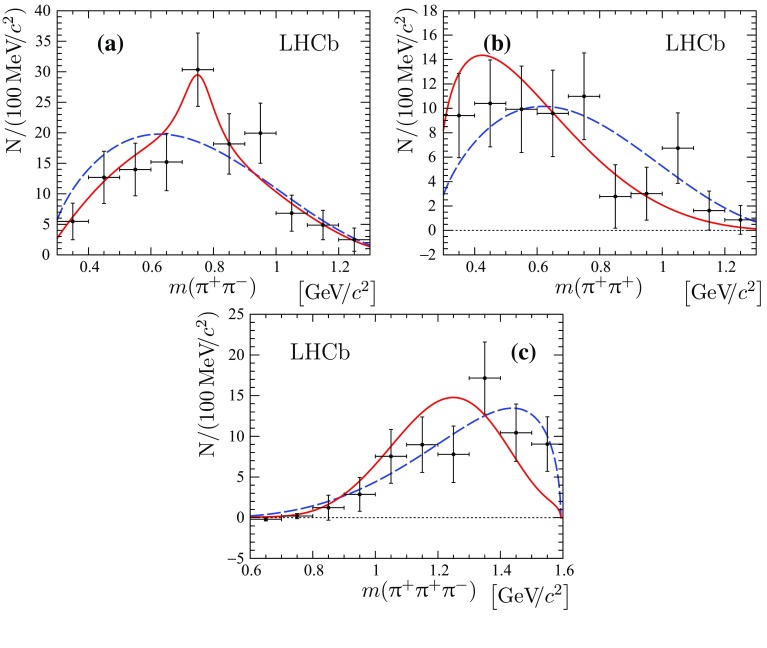
Distributions of **a** ${{\uppi } ^+} {{\uppi } ^-}$, **b** ${{\uppi } ^+} {{\uppi } ^+}$ and **c** ${{\uppi } ^+} {{\uppi } ^+} {{\uppi } ^-}$ masses in the ${{{\mathrm {B}} ^+}} \rightarrow {\uppsi {(2\mathrm {S})}} {{\uppi } ^+} {{\uppi } ^+} {{\uppi } ^-}$ decay. The predictions from the factorisation-based model is shown by *solid* (*red*) *lines*, and the expectation from the phase-space model is shown by *dashed* (*blue*) *lines*

In a similar way intermediate light resonances are searched for in the three-pion system recoiling against ${\uppsi {(2\mathrm {S})}} \rightarrow {{\mathrm {J}/\uppsi }} {{\uppi } ^+} {{\uppi } ^-}$ in ${{{\mathrm {B}} ^+}} \rightarrow {\uppsi {(2\mathrm {S})}} {{\uppi } ^+} {{\uppi } ^+} {{\uppi } ^-}$ decays. The resonant structure is investigated in the ${{\uppi } ^+} {{\uppi } ^-}$, ${{\uppi } ^+} {{\uppi } ^+}$ and ${{\uppi } ^+} {{\uppi } ^+} {{\uppi } ^-}$ combinations. The distributions for these combinations of pions are compared with predictions of both the factorisation approach and a phase-space model, as shown in Fig. 5. The corresponding $\chi ^2/\mathrm {ndf}$ values are summarized in Table 3. Similarly to the non-resonant case, the prediction from the factorisation approach is found to be in somewhat better agreement with the data than that from the phase-space model.

## Efficiencies and systematic uncertainties

The two ratios of branching fractions defined in Eq. 1 are measured as

$$\begin{aligned} R_{5\pi }&= \dfrac{N_{{{\mathrm {J}/\uppsi }} 3{{\uppi } ^+} 2{{\uppi } ^-}}}{N_{{\uppsi {(2\mathrm {S})}} [\rightarrow {{\mathrm {J}/\uppsi }} {{\uppi } ^+} {{\uppi } ^-} ]{{\mathrm {K}} ^+}}} \times \dfrac{\upvarepsilon _{{\uppsi {(2\mathrm {S})}} [\rightarrow {{\mathrm {J}/\uppsi }} {{\uppi } ^+} {{\uppi } ^-} ]{{\mathrm {K}} ^+}}}{\upvarepsilon _{{{\mathrm {J}/\uppsi }} 3{{\uppi } ^+} 2{{\uppi } ^-}}}\\&\quad \times {\mathcal {B}} ({\uppsi {(2\mathrm {S})}} \rightarrow {{\mathrm {J}/\uppsi }} {{\uppi } ^+} {{\uppi } ^-}),\\ R_{{\uppsi {(2\mathrm {S})}}}&= \dfrac{N_{{\uppsi {(2\mathrm {S})}} [\rightarrow {{\mathrm {J}/\uppsi }} {{\uppi } ^+} {{\uppi } ^-} ]{{\uppi } ^+} {{\uppi } ^+} {{\uppi } ^-}}}{N_{{\uppsi {(2\mathrm {S})}} [\rightarrow {{\mathrm {J}/\uppsi }} {{\uppi } ^+} {{\uppi } ^-} ]{{\mathrm {K}} ^+}}}\\&\quad \times \dfrac{\upvarepsilon _{{\uppsi {(2\mathrm {S})}} [\rightarrow {{\mathrm {J}/\uppsi }} {{\uppi } ^+} {{\uppi } ^-} ]{{\mathrm {K}} ^+}}}{\upvarepsilon _{{\uppsi {(2\mathrm {S})}} [\rightarrow {{\mathrm {J}/\uppsi }} {{\uppi } ^+} {{\uppi } ^-} ]{{\uppi } ^+} {{\uppi } ^+} {{\uppi } ^-}}}, \end{aligned}$$

where $N_{X}$ represents the observed signal yield and $\upvarepsilon _{X}$ denotes the efficiency for the corresponding decay. The known value of $(34.46\,\pm \,0.30)\%$ [1] is used for the ${\uppsi {(2\mathrm {S})}} \rightarrow {{\mathrm {J}/\uppsi }} {{\uppi } ^+} {{\uppi } ^-}$ branching fraction.

The efficiency is determined as the product of the geometric acceptance and the detection, reconstruction, selection and trigger efficiencies. The efficiencies for hadron identification as a function of the kinematic parameters and event multiplicity are determined from data, using calibration samples of kaons and pions from the self-identifying decays ${{\mathrm {D}} ^{*+}} \rightarrow {{\mathrm {D}} ^0} {{\uppi } ^+}$ followed by ${{\mathrm {D}} ^0} \rightarrow {{\mathrm {K}} ^-} {{\uppi } ^+}$ [23]. The remaining efficiencies are determined using simulated events.

**Table 2:** The $\chi ^2$ per degree of freedom for the factorisation-based and phase-space models for the multipion system in non-resonant ${{\mathrm {B}} ^+} \rightarrow {{\mathrm {J}/\uppsi }} 3{{\uppi } ^+} 2{{\uppi } ^-}$ decays

Multipion system	Factorisation model	Phase-space model
${{\uppi } ^+} {{\uppi } ^-}$	0.7	2.6
${{\uppi } ^-} {{\uppi } ^-}$	2.8	3.7
${{\uppi } ^+} {{\uppi } ^+}$	1.7	4.2
${{\uppi } ^+} {{\uppi } ^+} {{\uppi } ^-}$	1.8	2.3
${{\uppi } ^+} {{\uppi } ^-} {{\uppi } ^-}$	2.8	5.0
${{\uppi } ^+} {{\uppi } ^+} {{\uppi } ^+}$	1.0	2.5
$2{{\uppi } ^+} 2{{\uppi } ^-}$	3.5	4.4
$2{{\uppi } ^+} {{\uppi } ^-}$	0.7	1.0
$3{{\uppi } ^+} 2{{\uppi } ^-}$	2.2	1.7

To determine the overall efficiency for the ${{\mathrm {B}} ^+} \rightarrow {{\mathrm {J}/\uppsi }} 3{{\uppi } ^+} 2{{\uppi } ^-}$ channel, the individual efficiencies for the resonant and non-resonant components are averaged according to the measured proportions found in the data,

$$\begin{aligned} k \equiv \dfrac{N_{{\uppsi {(2\mathrm {S})}} [\rightarrow {{\mathrm {J}/\uppsi }} {{\uppi } ^+} {{\uppi } ^-} ]{{\uppi } ^+} {{\uppi } ^+} {{\uppi } ^-}}}{N_{{{\mathrm {J}/\uppsi }} 3{{\uppi } ^+} 2{{\uppi } ^-}}} = 0.44\pm 0.06. \end{aligned}$$

The ratio *k* is calculated taking into account the correlation in the observed values in the numerator and denominator. The ratios of the efficiency for the normalization channel $\upvarepsilon _{{\uppsi {(2\mathrm {S})}} {{\mathrm {K}} ^+}}$ to the efficiencies for resonant, $\upvarepsilon _{{\uppsi {(2\mathrm {S})}} {{\uppi } ^+} {{\uppi } ^+} {{\uppi } ^-}}$, and non-resonant decays $\upvarepsilon _{{{\mathrm {J}/\uppsi }} 3{{\uppi } ^+} 2{{\uppi } ^-}, \mathrm {NR}}$, are determined to be

$$\begin{aligned} \dfrac{\upvarepsilon _{{\uppsi {(2\mathrm {S})}} {{\mathrm {K}} ^+}}}{\upvarepsilon _{{\uppsi {(2\mathrm {S})}} {{\uppi } ^+} {{\uppi } ^+} {{\uppi } ^-}}}= & {} 6.75\pm 0.13, \\ \dfrac{\upvarepsilon _{{\uppsi {(2\mathrm {S})}} {{\mathrm {K}} ^+}}}{\upvarepsilon _{{{\mathrm {J}/\uppsi }} 3{{\uppi } ^+} 2{{\uppi } ^-}, \mathrm {NR}}}= & {} 4.18\pm 0.05. \end{aligned}$$

The ratio of efficiencies for the normalisation channel to that of the ${{\mathrm {B}} ^+} \rightarrow {{\mathrm {J}/\uppsi }} 3{{\uppi } ^+} 2{{\uppi } ^-}$  mode is given by

$$\begin{aligned} \dfrac{\upvarepsilon _{{\uppsi {(2\mathrm {S})}} {{\mathrm {K}} ^+}}}{\upvarepsilon _{{{\mathrm {J}/\uppsi }} 3{{\uppi } ^+} 2{{\uppi } ^-}}}= & {} k\times \dfrac{\upvarepsilon _{{\uppsi {(2\mathrm {S})}} {{\mathrm {K}} ^+}}}{\upvarepsilon _{{\uppsi {(2\mathrm {S})}} {{\uppi } ^+} {{\uppi } ^+} {{\uppi } ^-}}} + (1-k)\\&\times \dfrac{\upvarepsilon _{{\uppsi {(2\mathrm {S})}} {{\mathrm {K}} ^+}}}{\upvarepsilon _{{{\mathrm {J}/\uppsi }} 3{{\uppi } ^+} 2{{\uppi } ^-},{\mathrm {NR}}}}= 5.31\pm 0.06. \end{aligned}$$

The statistical uncertainty in the ratio *k* is accounted for in the calculation of the statistical uncertainty for the ratio $R_{5\pi }$.

**Table 3:** The $\chi ^2$ per degree of freedom for the factorisation-based and phase-space models for the multipion system recoiling against ${\uppsi {(2\mathrm {S})}}$ in ${{\mathrm {B}} ^+} \rightarrow {\uppsi {(2\mathrm {S})}} {{\uppi } ^+} {{\uppi } ^+} {{\uppi } ^-}$ decays

Multipion system	Factorisation model	Phase-space model
${{\uppi } ^+} {{\uppi } ^-}$	0.5	1.3
${{\uppi } ^+} {{\uppi } ^+}$	0.8	0.7
${{\uppi } ^+} {{\uppi } ^+} {{\uppi } ^-}$	1.3	1.6

Since the decay products in the channels under study have similar kinematics, many systematic uncertainties cancel in the ratio (for instance those related to muon identification). The different contributions to the systematic uncertainties affecting this analysis are described below. The resulting individual uncertainties are presented in Table 4.

**Table 4:** Relative systematic uncertainties (in %) for the ratios of branching fractions. The total uncertainty is the quadratic sum of the individual components

Source	$R_{{\uppsi {(2\mathrm {S})}}}$	$R_{5\pi }$
Fit model	4.6	2.4
Decay model	5.9	1.1
Hadron interactions	$2\times 1.4$	$2\times 1.4$
Track reconstruction	1.9	1.8
Hadron identification	0.3	0.3
Size of the simulation sample	1.9	1.2
Trigger	1.1	1.1
${\mathcal {B}} ({\uppsi {(2\mathrm {S})}} \rightarrow {{\mathrm {J}/\uppsi }} {{\uppi } ^+} {{\uppi } ^-})$	0.9	–
Total	8.5	4.7

The dominant uncertainty arises from the imperfect knowledge of the shape of the signal and the background in the ${{\mathrm {B}} ^+}$ and $\uppsi {(2\mathrm {S})}$  mass distributions. The dependence of the signal yields on the fit model is studied by varying the signal and background parametrisations. The systematic uncertainties are determined for the ratios of event yields in different channels by taking the maximum deviation of the ratio obtained with the alternative model with respect to the baseline fit model. The uncertainty determined for $R_{{\uppsi {(2\mathrm {S})}}}$ and $R_{5\pi }$ is 4.6 and 2.4%, respectively.

To assess the systematic uncertainty related to the ${{\mathrm {B}} ^+} \rightarrow {{\mathrm {J}/\uppsi }} 3{{\uppi } ^+} 2{{\uppi } ^-}$  (${{\mathrm {B}} ^+} \rightarrow {\uppsi {(2\mathrm {S})}} {{\uppi } ^+} {{\uppi } ^+} {{\uppi } ^-}$) decay model used in the simulation, the reconstructed mass distribution of the three-pion (five-pion) system in simulated events is reweighted to reproduce the distribution observed in data. There is a maximum change in efficiency of 5.9% for the resonant mode and 4.7% for the non-resonant mode leading to a 1.1% change in the total efficiency, which is taken as the systematic uncertainty for the decay model.

Further uncertainties arise from the differences between data and simulation, in particular those affecting the efficiency for the reconstruction of charged-particle tracks. The first uncertainty arises from the simulation of hadronic interactions in the detector, which has an uncertainty of 1.4% per track [24]. Since the signal and normalisation channels differ by two tracks in the final state, the corresponding uncertainty is assigned to be 2.8%. The small difference in the track-finding efficiency between data and simulation is corrected using a data-driven technique [24]. The uncertainties in the correction factors are propagated to the efficiency ratios by means of pseudoexperiments. This results in a systematic uncertainty of 1.9 and 1.8% for the ratios of $R_{{\uppsi {(2\mathrm {S})}}}$ and $R_{5\pi }$, respectively.

The uncertainties on the efficiency of hadron identification due to the limited size of the calibration sample are also propagated to the efficiency ratios by means of pseudoexperiments. The resulting uncertainties are equal to 0.3% for both branching fraction ratios. Additional uncertainties related to the limited size of the simulation sample are 1.9 and 1.2% for $R_{{\uppsi {(2\mathrm {S})}}}$ and $R_{5\pi }$, respectively.

The trigger is highly efficient in selecting decays with two muons in the final state. The trigger efficiency for events with a ${{\mathrm {J}/\uppsi }} \rightarrow {\upmu ^+\upmu ^-}$ produced in beauty hadron decays is studied using data in high-yield modes and a systematic uncertainty of 1.1% is assigned based on the comparison of the ratio of trigger efficiencies for high-yield samples of ${{\mathrm {B}} ^+} \rightarrow {{\mathrm {J}/\uppsi }} {{\mathrm {K}} ^+}$ and ${{\mathrm {B}} ^+} \rightarrow {\uppsi {(2\mathrm {S})}} {{\mathrm {K}} ^+}$ decays in data and simulation [25].

## Results and summary

A search for the decay ${{\mathrm {B}} ^+} \rightarrow {{\mathrm {J}/\uppsi }} 3{{\uppi } ^+} 2{{\uppi } ^-}$ is performed using a data sample corresponding to an integrated luminosity of 3.0 fb$^{-1}$, collected by the LHCb experiment. A total of $139\pm 18$ signal events are observed, representing the first observation of this decay channel. Around half of the ${{\mathrm {B}} ^+}$ candidates are found to decay through the $\uppsi {(2\mathrm {S})}$  resonance. The observed yield of  ${{\mathrm {B}} ^+} \rightarrow {\uppsi {(2\mathrm {S})}} {{\uppi } ^+} {{\uppi } ^+} {{\uppi } ^-}$  decays is $61\pm 10$ events, which is the first observation of this decay channel.

Using the ${{\mathrm {B}} ^+} \rightarrow {\uppsi {(2\mathrm {S})}} {{\mathrm {K}} ^+}$ decay as a normalisation channel, the ratios of the branching fractions are measured to be

$$\begin{aligned} R_{5\pi }&= \dfrac{{\mathcal {B}} ({{\mathrm {B}} ^+} \rightarrow {{\mathrm {J}/\uppsi }} 3{{\uppi } ^+} 2{{\uppi } ^-} )}{{\mathcal {B}} ({{\mathrm {B}} ^+} \rightarrow {\uppsi {(2\mathrm {S})}} {{\mathrm {K}} ^+} )} \\&= (1.88\pm 0.17\pm 0.09)\times 10^{-2}, \\ R_{{\uppsi {(2\mathrm {S})}}}&= \dfrac{{\mathcal {B}} ({{\mathrm {B}} ^+} \rightarrow {\uppsi {(2\mathrm {S})}} {{\uppi } ^+} {{\uppi } ^+} {{\uppi } ^-} )}{{\mathcal {B}} ({{\mathrm {B}} ^+} \rightarrow {\uppsi {(2\mathrm {S})}} {{\mathrm {K}} ^+} )} \\&= (3.04\pm 0.50\pm 0.26)\times 10^{-2}, \end{aligned}$$

where the first uncertainties are statistical and the second are systematic. The ratio $R_{5\pi }$ contains also the contribution from ${{\mathrm {B}} ^+} \rightarrow {\uppsi {(2\mathrm {S})}} [\rightarrow {{\mathrm {J}/\uppsi }} {{\uppi } ^+} {{\uppi } ^-} ]{{\uppi } ^+} {{\uppi } ^+} {{\uppi } ^-}$ decays.

The multipion distributions in the ${{\mathrm {J}/\uppsi }} 3{{\uppi } ^+} 2{{\uppi } ^-}$ final state (vetoing the $\uppsi {(2\mathrm {S})}$ meson contribution) and in the ${\uppsi {(2\mathrm {S})}} {{\uppi } ^+} {{\uppi } ^+} {{\uppi } ^-}$ final state are studied. A structure which can be associated to the $\uprho ^{0}$ meson is seen in the ${{\uppi } ^+} {{\uppi } ^-}$ combinations of the ${{\mathrm {J}/\uppsi }} 3{{\uppi } ^+} 2{{\uppi } ^-}$ final state. The multipion distributions are compared with the theoretical predictions from the factorisation approach and a phase-space model. The prediction from the factorisation approach is found to be in somewhat better agreement with the data than the prediction from the phase-space model.
